# Management of COVID‐19 and vaccination in Nepal: A qualitative study

**DOI:** 10.1111/hex.13732

**Published:** 2023-02-16

**Authors:** Alisha Karki, Barsha Rijal, Bikash Koirala, Prabina Makai, Pramod KC, Pratik Adhikary, Saugat Joshi, Srijana Basnet, Sunita Bhattarai, Jiban Karki

**Affiliations:** ^1^ PHASE Nepal Bhaktapur Nepal

**Keywords:** COVID, management, Nepal, rural, urban, vaccination

## Abstract

**Objective:**

The aim of this research is to investigate the perspective of citizens of Nepal on the management COVID‐19, the roll‐out of the vaccine, and to gain an understanding of attitudes towards the governments' handling of the COVID‐19 pandemic.

**Method:**

A qualitative methodology was used. In‐depth interviews were conducted with 18 males and 23 females aged between 20 and 86 years old from one remote and one urban district of Nepal. Interviews were conducted in November and December 2021. A thematic approach was used to analyse the data, utilising NVivo 12 data management software.

**Result:**

Three major themes were identified: (1) Peoples' perspective on the management of COVID‐19, (2) people's perception of the management of COVID‐19 vaccination and (3) management and dissemination of information. It was found that most participants had heard of COVID‐19 and its mitigation measures, however, the majority had limited understanding and knowledge about the disease. Most participants expressed their disappointment concerning poor testing, quarantine, vaccination campaigns and poor accountability from the government towards the management of COVID‐19. Misinformation and stigma were reported as the major factors contributing to the spread of COVID‐19. People's knowledge and understanding were mainly shaped by the quality of the information they received from various sources of communication and social media. This heavily influenced their response to the pandemic, the preventive measures they followed and their attitude towards vaccination.

**Conclusion:**

Our study concludes that the study participants' perception was that testing, quarantine centres and vaccination campaigns were poorly managed in both urban and rural settings in Nepal. Since people's knowledge and understanding of COVID‐19 are heavily influenced by the quality of information they receive, we suggest providing contextualised correct information through a trusted channel regarding the pandemic, its preventive measures and vaccination. This study recommends that the government proactively involve grassroots‐level volunteers like Female Community Health Volunteers to effectively prepare for future pandemics.

**Patient and Public Contribution:**

This study was based on in‐depth interviews with 41 people from diverse socioeconomic backgrounds. This study would not have been possible without their participation.

## 1 INTRODUCTION

COVID‐19 was declared a pandemic by the World Health Organization (WHO) on 11 March 2020.^1^ Since the outbreak, the WHO urged governments to prioritise their actions in response to the COVID‐19 infection. Beyond the disease itself, unprecedented social and economic hardship has been experienced across the globe due to this infection.^2^ Furthermore, emerging new variants of COVID‐19, causing subsequent waves of infection have caused concern worldwide, hastening the urgency for disease control, and the necessity for a plan to facilitate the end of the pandemic.^3^

The first step to controlling a pandemic like COVID‐19 is to stop the spread of infection. This responsibility falls under the preview of state governments. Good governance is paramount towards the effective management of COVID‐19.^4^ Many countries have adopted preventive measures such as social distancing, issuing advice on the use of hand sanitizers and wearing masks to curb the spread of the virus. After a continuous rise in cases, a rigorous lockdown was imposed to stop the spread of COVID‐19 in countries including Italy, Spain, France and the United Kingdom.^5^, ^6^ The government of Nepal (GoN) also imposed a complete lockdown on 24 March 2020, during the first phase of COVID‐19.^7^ The effectiveness of wearing masks, and other preventative measures have been proven to slow the spread of infection,^8^ and it is, therefore, essential for the government to educate the public on these health messages.

There have been 1,000,631 confirmed cases of COVID‐19 with 12,019 deaths with 5,958,956 polymerase chain reaction(PCR) tests as of 2 November 2022, in Nepal.^9^ Nepal has been responding to the pandemic through the implementation of public health prevention and hospital‐based interventions. Key interventions such as management of quarantine, screening and testing have been carried out. Dissemination of information related to COVID‐19 to the public, and managing vaccination campaigns were conducted to slow the spread of infection.^4^ Different management committees and task teams were also formed to minimise the adverse impact of COVID‐19 in Nepal.^10^ However, some of these committees were criticised for not being able to effectively implement such preventive strategies. Some academics have expressed the opinion that the potential risk of coronavirus transmission at the community level was not taken seriously in Nepal.^11^

Preventive initiatives, mass testing of COVID‐19 and quarantine measures are all equally important interventions in stopping the spread of the disease.^12^ Mass testing helps people to determine COVID‐19 infection regardless of symptom status, and being at risk of spreading the infection. Several international studies have found a reluctance towards COVID‐19 testing due to long queues, exposure risks and late reporting.^13^ Concerns were raised regarding testing disparities between rural and urban residents in Florida.^14^ Despite several efforts, Nepal was also not able to conduct sufficient diagnostic tests, and perform timely contract tracing in the initial phase of COVID‐19 transmission. COVID‐19 testing sites were limited by higher costs and longer time for test results.^2^ The lack of coordination and blame games among different stakeholders were found to be a prominent obstacle towards the management of COVID‐19 testing and quarantine services in Nepal. During the first wave, the authorities failed to manage effective provision for testing, isolation and quarantine services despite these being the heart of effective public health measures against COVID‐19.^11^ However, the government corrected the loopholes from the first wave (2020), which resulted in a contrasting response strategy during the second wave (2021).^11^

Besides several preventive measures, the development of a vaccine against COVID‐19 is considered a crucial moment in the efforts to curb disease spread and resume a normal life.^15^ Nepal began its first vaccination campaign in January 2021, with donations received from India.^16^ The GoN succeeded in managing vaccines through strong bilateral coordination, during global concern around the scarcity of vaccines.^11^ As of 13 September 2022, a total of 53,506,207 vaccines have been administered, accounting for approximately 88.9% of the total population, with 79.5% and 76.5% coverage of the first and second doses, respectively.^9^ This signifies remarkable effort and achievement for a resource‐limited country such as Nepal. The most high‐risk and vulnerable groups were prioritised for vaccination following the prioritisation protocol of WHO. Some concern was expressed on the way in which vaccination centres were managed, with particular concerns about the spread of infection due to crowding in vaccination centres.^2^ However, despite several challenges, the GoN has fully vaccinated 76.5% of the total population.^17^

It is imperative that governments are prepared for future waves of COVID‐19. It is essential that the management of pandemic preparedness and response is organised and sustainable.^18^ The effective management of COVID‐19 is the most urgent health issue globally today, and to this end, much research has been conducted to assess public knowledge and attitudes perceptions towards the disease.^19^, ^20^ However, to our knowledge, the management of COVID‐19 and government effectiveness in the management of COVID‐19 vaccines at the community level has not been studied yet in Nepal. Therefore, this study aims to gain the perspectives of the public towards the management of COVID‐19 and its vaccination in Nepal. This research will be useful for developing strategies and formulate contextualised plans and policies based on urban and rural settings in the event of future outbreaks, if any. Questions this study aims to answer:

1. How is COVID‐19 being managed in the rural and urban communities of Nepal?
2. What is the people's perspective towards the management of COVID‐19 and its vaccination?

## 2 METHODS

### 2.1 Study design

A qualitative research methodology was used^21^ to assess the perspective of people towards the management of COVID‐19 and vaccination in rural and urban areas of Nepal. The study was guided and presented in accordance with the Consolidated Criteria for Reporting Qualitative research Checklist.^22^

### 2.2 Research participants

In‐depth interviews^23^ (IDIs) were conducted with members of the public residing in the rural and urban areas of Soru Rural Municipality (RM) and Suryabinayak Municipality in Mugu and Bhaktapur districts of Nepal, respectively. All the participants were purposively selected^24^ based on the following inclusion criteria: (a) Participants living in the selected municipalities. (b) Eighteen years of age or older. (c) The ability to speak in the interview and willingness to participate in the study. Similarly, we also considered the diversity of participants based on age, gender, educational level and COVID‐19 vaccination status living in rural and urban communities of Nepal.

### 2.3 Data collection

Semistructured interview guidelines were used to conduct IDIs.^24^ All the interviews were conducted between November and December 2021. Interview guidelines were developed in the Nepali language and then translated into English. We included questions on the interviewees' sociodemographic characteristics and their knowledge, attitudes and perceptions of COVID‐19, and in particular, their opinions on the government's role in the management of testing and vaccination against the disease. Face‐to‐face interviews were conducted with the participants at their place of convenience, mostly at their homes and field, with the researcher, the interviewee and no‐one else present. Before commencing the interviews, the purpose of the study was explained to the participants, as well as the benefits and possible harms. Participants were given an information sheet, and their right to withdraw from the study at any point was emphasised. Participants were also asked to consent, verbally and in written form, to participate and to digitally record the interview. All of the interviews were audio‐recorded on an encrypted digital recorder and stored on a password‐protected computer. The audio‐recorded interviews were transcribed into Nepali and further translated into English. All of the personal identifiers of participants were replaced with unique codes. The confidentiality and anonymity of the research participants were maintained at all stages of the study. All necessary safety precautions were adhered to during the entire process of the interview, considering the risk of the COVID‐19 pandemic.

The data collection tool was pretested and necessary changes were made before the data collection. Participants were interviewed on one occasion only, and transcripts were not returned to interviewees for comments or clarifications. Among the participants approached for conducting IDIs, two of them declined to participate due to their personal work. We piloted four interviews before conducting the data collection at the study sites.

### 2.4 Data analysis

A thematic approach based on the work by Clarke et al.^25^ was used to analyse the qualitative data. In the first step, all of the recorded interviews were carefully listened to multiple times, and then transcribed verbatim and translated into English, to ensure familiarity with the contents. Other co‐authors collaborated to identify the commonalities and differences in the interview transcripts and worked to develop an initial set of themes. Potential themes were reviewed and named, ensuring coherence and a good representation of data. After thematic identification, the first and second authors completed open coding manually with five of the interview transcripts chosen based on the representativeness of the entire data set. The first author refined the coding framework and applied this framework to the rest of the data set. We exported the framework matrix as a spreadsheet and then summarized it into relevant themes. Any alterations to the themes or codes were discussed collectively and agreed upon by the research team. The codebook was finalised through regular team meetings during the data analysis process. Five researchers coded the entire data set and 10 interview transcripts were double‐coded. Similarly, five researchers were involved in generating themes. We used NVivo 12 (Version 12 pro; QSR International),^26^ a qualitative data management software for codebook management and data analysis.

### 2.5 Reflexivity

All interviewers are from public health and medical backgrounds and have prior experience in conducting qualitative interviews. The interviewers built rapport with the participants and endeavoured to be neutral throughout the interview, to avoid researcher bias and facilitate the free flow of opinions from the participants. Overall, as a team, we presented a different perspective and contextual knowledge which strengthened the quality and validity of our study. Four researchers (A. K., B. R., S. J. and S. B.) designed the study proposal and prepared interview guidelines with the support of (B. K., P. K. C., P. A., J. K. and P. M.). Four researchers (A. K., B. R., S. J. and S. B.) were involved in the data collection. Five of the researchers (A. K., B. R., B. K., S. J. and S. B.) were involved in data analysis and manuscript preparation. Other members of the writing team contributed to drafts and to refining the manuscript.

## 3 RESULTS

Table 1 contains the demographic information of the 41 respondents who voluntarily participated in this study. Out of the 41 selected participants, 23 were female and 18 were male. The age range of the participants was from 20 to 86 years old. Most (11) of the respondents were illiterate and did not receive any form of formal or informal education. Of the 41 respondents, 21 were from urban areas, while 20 were from rural locations. At the time of the study, 4 respondents were unvaccinated against COVID‐19, while the remaining 37 were vaccinated. To get a diverse viewpoint, both vaccinated and unvaccinated participants were included in the study. The average length of interviews was 30 min, and field notes were also taken. After data saturation was obtained and no new information was generated, we stopped recruiting participants for the interview.

**Table 1:** Demographic characteristics of the respondents.

S. No.	Demographic characteristics	*N* (%)
1.	Age (mean ± SD)	37.93 ± 14.67
2.	Sex	
	Female	23 (56)
	Male	18 (44)
3.	Education	
	Illiterate	11 (26.8)
	Primary	1 (2.4)
	Secondary	10 (24.3)
	Intermediate	9 (21.9)
	Bachelor	6 (14.6)
	Masters	4 (9.7)
4.	Ethnicity	
	Brahmin/Chhetri	30 (73.1)
	Janajati	6 (14.6)
	Thakuri	4 (9.7)
	Dalit	1 (2.4)
5.	Residence	
	Rural	21 (51.2)
	Urban	20 (48.7)
6.	Vaccination status	
	Vaccinated	37 (90.2)
	Unvaccinated	4 (9.7)

### 3.1 Qualitative findings

Findings have been summarized into three major themes: (i) Peoples' perspective on the management of COVID‐19, (ii) peoples' perception of the management of COVID‐19 vaccination and (iii) management and dissemination of information. In the first theme, we have included responses regarding the preventive measures participants took to avoid COVID‐19, as well as the management of confirmed and suspected cases, COVID‐19 testing and quarantine. Similarly, the second theme includes participants' perception of the management of access to COVID‐19 vaccines, their trust and awareness regarding COVID‐19 vaccination and overall management of COVID‐19 vaccination in their area. The third theme contains participants' perspectives on the role and influence of social media on COVID‐19 and vaccination in both urban and rural areas.

#### 3.1.1 Theme 1: Peoples' perspective on the management of COVID‐19

##### Following preventive measures

Participants were aware of preventive measures such as wearing masks, washing their hands, using hand sanitizers and keeping a physical distance to prevent infections, but such safety measures were only followed in larger meetings or gatherings, not on a regular basis. In rural communities of Nepal, mass media like radio and FM were used by the people for information regarding the preventive measures for COVID‐19. Similarly, in urban areas, people generally had access to personal protective equipment such as masks, sanitizers and soaps. However, as time passed, the practice of these measures shifted from more cautious adoption in the beginning to less serious adherence to these practices.

People follow the safety measures only during the meetings in the rural municipality and other gatherings. The health workers follow it even now. Other than that, people do not use masks and sanitizers in the present time. (SR_19)

There were only a few households that gave continuous special attention and care to preventive measures because they wanted to safeguard the health of small children in the family.

Yes, I think I am following the protocols more closely than other members of my family because I have a baby and they have less immunity to fight against any kind of disease. (SB_13)

In rural areas, people had limited access to hygiene products such as masks, soaps and sanitizers, and used them only when they were freely distributed, indicating both affordability and access problems.

People wore masks when they were distributed by the local government, but they didn't buy them by themselves after that and also didn't continue wearing them. (SR_20)

To control the spread of COVID‐19, quarantine centres were also available in both rural and urban areas, specifically targeting returnee migrants. However, as time passed, such practices were not followed strictly. Participants from urban areas voiced their concerns that quarantine centres were not properly managed and due to over‐crowding, their use posed a high risk of infection.

It is good that the government tried to manage quarantine, but most of the people complained that the management was not nice. COVID‐19 was most commonly transmitted in quarantined areas. It is good that the government managed quarantine, but I think it was not effective. (SB_4)

Participants in rural areas stated that quarantine centres were soon abandoned, as, in addition to being crowded and poorly managed, there was not sufficient food available for residents. In rural areas, respondents voiced that they opted to quarantine at home instead.

At that time, the local government assigned their health workers to quarantine centres. There was a crowd, as more people had to adjust in a single room. It might be due to insufficient space. I think food and other basic needs are managed at the local level. I heard that some of them were trying to leave the quarantine centres as they were not providing good food, shelter, or fear of getting infection from another person. (SR_11)

3.1.1.1

####### Managing confirmed and suspected cases

In both rural and urban areas, the management of positive and suspected cases of COVID‐19 with symptoms was primarily done at home, except for emergency cases. People turned to home remedies in large numbers, reviving traditional tonics made from ginger, turmeric and cumin to treat flu‐like symptoms. A less common herb, known as Gurjo/Guduchi (heart‐shaped moonseed) was also extensively used, as respondents’ voices that they believed would supposedly reduce the chances of COVID‐19 complications by strengthening immunity. Participants disclosed that those who had enough rooms and a separate toilet were able to isolate themselves properly, in comparison to those who lived in small and shared spaces. Likewise, it was also indicated that hospitals were reluctant to admit COVID‐19 suspected patients and suggested that they stay at home unless there was a medical emergency. However, the work of the government hospital in providing free‐of‐cost services to cure COVID‐19 was well noted and appreciated by participants.

As I saw in the news, oxygen cylinders were managed for the COVID‐infected people as per requirements by the government. But those who didn't need oxygen and whose saturation didn't drop beyond the minimum stayed at home and took the required precautions. People were likely to drink Gurjo water (a medicinal herb) and boiled hot water during that time. Mostly, the hospital hesitated to take the cases of COVID‐19 during that time. (SB_1)

It was found that the onset of the COVID‐19 pandemic triggered feelings of fear and panic among our participants, leading to further stigmatisation of the disease and those infected. This stigmatisation led people to hide their infections and, in some cases, neglect to test, to evade discriminatory treatment in society. In such cases, instead of isolating, people continued their daily activities and contributed to the transmission of the disease in the community. This posed a challenge in controlling the infection's spread, thus creating a huge loophole in tracking and managing the infected and suspected cases.

I think one of our neighbours was infected by corona before me. But they didn't tell us that they were infected. People didn't inform other people about the COVID infection during those times. We didn't even tell anyone that I was infected by Covid‐19. (SB_5)

###### Management of COVID‐19 testing

Participants shared their opinions on the management of the pandemic, stating that the COVID‐19 test was inefficient in the beginning, but became gradually more accessible, especially in urban areas. Early in the pandemic, there were very few government labs doing PCR testing, which gradually changed when the testing equipment became more widely available, and private clinics and hospitals began to conduct such testing.

Now it is not that far to travel for PCR testing. It might be one kilometre away from this place. If people paid money and went to private clinics for their tests, then it was easy, but at the government testing site, the public had to face a long queue and it was not properly managed at all. (SB_8)

In rural areas, PCR testing facilities were rare, meaning that people had to travel to the District Hospitals to undergo testing, when facilities were in place. Such travel incurred a significant financial burden, and was time consuming, requiring the hiring of a jeep as well as hours of walking on foot. Testing of suspected cases was only made locally possible with the availability and use of the antigen test. However, the local test campaigns were short‐term, with all the test facilities concentrating on the RM centres eventually.

It takes Rs. 500 in the jeep to reach the testing site. It takes about 6 h to get there on foot. (SR_20)

It was placed in a nearby school for a few days. After that, it was shifted back to the rural municipality. (SR_10)

#### 3.1.2 Theme 2: Peoples’ perception of the management of COVID‐19 vaccination

##### Managing access

The participants in the study had difficulty accessing COVID‐19 vaccinations, indicating discrepancies in vaccine distribution and management. Initially, vaccination was provided to health workers and frontline workers such as security personnel, and politicians. Study participants voiced appreciation for these measures, but concerns were espoused regarding the manner in which the vaccination programme was managed and rolled out. Several participants responded that there was initially a scarcity of vaccines, which was only within the reach of high‐ranking officials, politicians or those with good connections. Furthermore, there were also complaints and doubts over the delayed and inequitable distribution of vaccines, especially in rural areas, signalling a gap and an inefficient supply of available vaccines. Some health professionals shared that they also struggled to access vaccines.

The vaccine was given to health workers based on their age group by the government. That was very nice in my opinion. So, I would thank the government for that. (SR_17)

Only those with connections to health workers, as well as those with power and connections, could easily obtain vaccines. (SB_13)

People who lived in the rural areas were deprived of vaccination. Urban areas were prioritised first. (SB_10)

##### Peoples' trust and awareness regarding vaccination

Study participants in both rural and urban areas voiced their opinions that there is a need to raise awareness about vaccines and the importance of vaccination. This is because many people declined the vaccine, even in situations where it was offered free of charge contrary to participants' good level of knowledge regarding the disease, there were suspicions and fears regarding the vaccines. To combat these anxieties, accurate, positive information dissemination is required to educate the public. While many respondents had trust in vaccines, the majority of community members were doubtful of their effectiveness as well as wary of the risk of complications.

Even in my home, my parents are not educated. It is our responsibility to make people aware of the importance of vaccination. If educated people like us get vaccinated, then other people will follow in our footsteps. There were many people who didn't want to get vaccinated, but after seeing other people, they slowly decided to get vaccinated. (SR_10)

##### Management of COVID‐19 vaccination

The majority of participants reported the vaccination campaign to be inefficient and poorly managed. People from both the rural and urban areas mentioned that they had to travel to vaccination centres multiple times to seek vaccination. Additionally, respondents from rural areas expressed that the process has been slow and the vaccines sent to the villages were inadequate, indicating inadequacy in supply chain management.

For two days, I was in a queue and only, after so much struggle, did I get vaccinated. Other people faced a similar issue as well. In the beginning, it was not easy to get vaccinated, and the government didn't manage properly. (SB_09)

I am not satisfied with the local government as it has not been able to provide the vaccine in the required amount and on time. So, if every person got the opportunity to get vaccinated on time, then it would be better for everyone. (SR_18)

Regardless of these challenges, many participants felt that the GoN managed the COVID‐19 vaccination programme efficiently, despite its status as a resource‐limited country. The COVID‐19 vaccination programme was prioritised for frontline health workers, frontline security personnel and the elderly population which was appreciated by the majority of our participants.

It is good that the government has provided vaccines for frontline workers and vulnerable populations. I think the government has provided this service according to different categories to manage it in a systematic way. It is a good way to provide the vaccine. (SB_12)

#### 3.1.3 Theme 3: Management and dissemination of information

Participants from both rural and urban areas reported that there was a clear flow of disease limitation guidance, particularly regarding prevention measures, screening, isolation and treatment, which was well received by the public, resulting in a high level of awareness. The use of mass media communication tools such as FM radio appears to have been utilised effectively to communicate health messages. On the other hand, suspicions still prevailed regarding the effect and efficacy of the vaccination, even when a large number of people had already been vaccinated. The following statements illustrate the range of health messages participants received:

For people like us who work and go to other places, it's easy‐to‐get information. Other people rely on the radio and their friends for such information. Information like drinking hot water, keeping ourselves warm, avoiding going to the crowd, and other safety measures were heard from the radio. (SR_8)

My neighbours were in confusion about whether to get vaccinated or not. They also heard that people who were vaccinated against COVID had some side effects, so they were confused about whether to get vaccinated. (SB_01)

There was delayed information, lack of information and misinformation regarding COVID‐19 and its vaccination according to a minority of the study participants. The information some participants received was not complete.

We were a bit late in receiving information regarding the COVID‐19 vaccine. If people were informed about the benefits and possible side effects of the vaccine earlier to vaccination, then more people would have chosen to get vaccinated. (SB_06)

People who had access to the internet, primarily those in urban communities, were the ones being influenced by misinformation shared on social media platforms. Social media played a major role in spreading hoax news about the vaccine and its effect. A portion of study participants voiced that they were reluctant to get vaccinated, mainly due to the rumours of ineffectiveness and possible side effects like fever and body pain that were circulated via Facebook.

I heard that people who get vaccinated could face serious consequences. If they are infected with COVID‐19 then they could even have to be admitted to the ICU and coma. In the initial phase of vaccination, I didn't opt for it due to such rumours. But later when the situation began to normalize, I received the vaccine against COVID‐19. (SB_08)

In rural communities like Soru RM of Nepal, people have limited access to other forms of mass media like television, the internet and mobile phones. In these communities, radio stations are the primary source of information. The reliance on radio, and the relative absence of use of social media means that participants in rural areas were found to be less affected by the untrue rumours and misinformation circulated through social media.

We heard many rumours in our village as well but such rumours didn't have much effect among people regarding the vaccination program. Here, we mostly rely on local radio channels and FM programs for news and information, so I don't think that anybody in this community has a wrong impression of vaccination against COVID‐19. (SR_10)

## 4 DISCUSSION

This study was conducted to gain an understanding of the perspective of people living in rural and urban communities, towards the management of COVID‐19 and its vaccination within Nepal. The study findings show that most participants from both urban and rural areas were well aware of management‐related aspects of COVID‐19, from preventive measures to vaccination. However, stigmatisation, mismanagement of testing and quarantine centres and vaccine hesitancy due to misinformation were prevalent at the community level. These factors led to management aspects not being taken seriously, especially during the latter phase of the pandemic. A similar study in Nepal also highlighted issues like the carelessness of individuals towards COVID‐19 management and the government's inability to manage testing, quarantine, and vaccination. These were found to be the major obstacles in the effective management of COVID‐19 and vaccination.^27^ International research^28^ has highlighted the problems of improper management of resources and equipment, lack of guidelines for contact tracing and patient flow management, which are not unique to Nepal. The lack of proper management of isolation and quarantine centres has caused a significant spike in the cases of COVID‐19, especially in rural areas of Nepal, where the majority of cases were imported by migrant workers from India.^6^ This demonstrates a lack of effective planning, preparedness and coordination of relevant authorities in response against the COVID‐19 pandemic.

The study found that, aside from emergency cases that required hospital treatment, the majority of positive and suspected cases were managed at home using traditional/home remedies, as individuals preferred staying at home rather than in quarantine centres. This was due to the poor condition of the majority of quarantine centres, fear of stigmatisation and prejudice of uncertainty about the pandemic situation at the community level. These results echo the findings of international research identifying that fear of stigma and discrimination, leads people to attempt to hide cases of suspected infection.^29^ Similar studies conducted in Malaysia^30^ and Ethiopia^31^ found that people who had tested positive for COVID‐19 were isolated, labelled and blamed by their peers, including healthcare providers. This led people to hide their status, which eventually spread the disease in the community. This demonstrates the way in which prejudice in the community can lead to a delay in individuals seeking testing and treatment essential for their own health, and the safeguarding of the community. Various media reports also indicated that people in Nepal were reluctant to stay in quarantine centres.^17^ This implies the inefficiency of the government towards the management of COVID‐19, which was mainly due to overcrowding, lack of basic facilities to maintain personal hygiene and sanitation, and a poor living environment.^32^ These results reinforce the findings of further research conducted in South Asia, that quarantine centres were managed poorly, with an insufficient supply of food and water, a lack of healthcare facilities and poor sanitation.^33^

COVID‐19 testing facilities were rarely available during the first phase of the pandemic, during which testing was only within the reach of people within the urban population. However, the financial burden of testing was passed onto the individual and proved unaffordable for the vast majority of the general population. In Nepal, people were found to be reluctant to test themselves against COVID‐19.^34^ This mirrors the findings of research conducted overseas, including in the United Kingdom.^35^ The refusal of at‐risk individuals to test drastically impedes effective contact tracing and presents a barrier to control of the disease at the community level. Additionally, this contributes to delays in diagnosis and appropriate isolation of suspected cases, exacerbating the difficulties further.

In terms of vaccination, study participants voiced their discontent about the distribution of vaccines and management of vaccination centres. Some respondents relayed rumours spread through a variety of communication channels. Misinformation created due to health‐related uncertainty and spread via unreliable sources of information such as social media platforms present challenges to a vulnerable healthcare system. Inadequate pandemic preparedness and overall weak institutional infrastructure in the health sector contributed to a shortfall of public trust towards the health system of Nepal. The results indicate that participants supported the measure of frontline workers being given priority in vaccination against COVID‐19, however, they were frustrated with how it was politicised at the local level, with vaccination priority being given to those in government roles. Research conducted in Nepal recommends that legislators should focus on more effective management of logistics, distribution, and delivery systems.^36^ An essential lesson is that the availability of vaccines alone is not conducive to disease control. Vaccine rollout must be accompanied by accessibility at community level, and accurate, coherent information dissemination to the public, utilising reliable sources, in order for the public to understand the relevance and importance of their role in getting vaccinated to slow the spread of the disease.^37^

Social media is a tool that has spread both positive and negative information as COVID‐19 triggered a global infodemic. This has undermined public trust in government messages, and lead to serious challenges for virus containment, both of which have outlasted the coronavirus pandemic.^38^ This study has also identified that social media platforms have had a great role to play in shaping people's behaviour and attitude towards COVID‐19 and vaccination, especially in urban areas, where the majority of people have access to media via the internet. Research conducted in Pakistan^38^ highlights the correlation between excessive usage of social media to gather information, and change in COVID‐19‐related health‐related behaviours. Information disseminated via these media streams has impacted health behaviours at the individual level, with some utility, particularly early in the pandemic, when screening, testing and vaccination measures were not available. However, the role of social media in spreading negative messages about the vaccine, and its contribution to vaccine hesitancy, and reluctance to adopt positive health behaviours, cannot be ignored.

Trust in government and vaccine assurance were to be key mediators across COVID‐19 information in the media and vaccine reluctance. The terrain and topography of Nepal mean that the geographical disparity between urban and rural settings is huge. At the community level, the risk of contracting the infection and spreading COVID‐19 was not taken seriously in Nepal, especially in the later phase. If basic prevention strategies are not adopted seriously, then the pandemic will deepen. Prevention and control messages could be disseminated through awareness campaigns designed to encourage people to follow preventive measures, including getting vaccinated against COVID‐19. The government has 2 years of experience in the management of COVID‐19 and needs to build on this by incorporating experts in the management of COVID‐19. Nonetheless, we cannot deny that despite having various challenges in COVID‐19 prevention and control in Nepal, there were noteworthy efforts undertaken by the government.

## 5 STRENGTH AND LIMITATIONS

The findings represent the voices and views of the public from both rural and urban communities of Nepal regarding the management of the COVID‐19 pandemic and vaccination conducted by the government. The results discussed in this study are a useful addition to the international body of research on COVID‐19 and its control. The lessons learned can be useful in managing future pandemics. This research will be useful to policy makers and implementers in evidence‐based decision making. However, since this study was conducted only in two districts in Nepal, the findings from this study might not be representative of the perspective of people from elsewhere in the country. Similarly, overrepresentation of participants from the Brahmin and Chhetri ethnic groups might have affected the results of our study. In addition, this study investigates only the perspective of members of the public towards the management of COVID‐19 and its vaccination. This does not include perspectives from government‐level officials or other professionals involved in the management of the pandemic, which would be a useful topic for further research.

## 6 CONCLUSIONS

The study concludes that testing, quarantine centres and vaccination campaigns were poorly managed in both urban and rural settings in Nepal. However, the government's effort to manage the vaccines for frontline workers was highly appreciated by the public. It has been demonstrated that individuals' knowledge and understanding of COVID‐19 are heavily influenced by the quality of information they receive. For this reason, it is suggested that contextualised factual information is provided, through a trusted channel, regarding health messages, related to and the pandemic, its preventive measures, and vaccination. It is essential that national government bodies collaborate with local government agencies and mobilise community volunteers to work together proactively in the implementation of mitigation measures.

## AUTHOR CONTRIBUTIONS

Saugat Joshi, Alisha Karki, Barsha Rijal, Srijana Basnet and Jiban Karki conceptualised and designed the study. Saugat Joshi, Alisha Karki, Barsha Rijal and Srijana Basnet contributed to the literature review and data collection. Saugat Joshi, Alisha Karki, Barsha Rijal, Srijana Basnet, Bikash Koirala, Pratik Adhikary, Pramod KC, Prabina Makai and Jiban Karki contributed to data analysis and data interpretation. Saugat Joshi, Alisha Karki, Barsha Rijal, Srijana Basnet and Jiban Karki wrote the first draft and received input from Bikash Koirala, Pramod KC, Prabina Makai, Sunita Bhattarai and Pratik Adhikary during revision. All authors performed draft editing and final draft preparation. All authors have read and approved the final manuscript.

## CONFLICT OF INTEREST STATEMENT

The authors declare no conflict of interest.

## ETHICS STATEMENT

The ethical approval was obtained from the ethical review board of the Nepal Health Research Council, Nepal (approval number 625/2021P, ref no 1101, 9 November 2021) for this study. Participants were informed about voluntary participation and their right to withdraw at any time from the interview. The objective of the study was clearly mentioned to the participants. Informed and written consent was obtained from the participants before the interview. The interview was conducted according to the time and preferences of the participants.

## Data Availability

The data that support the findings of this study are available from the corresponding author upon reasonable request.

## References

[1] Zheng J . SARS‐CoV‐2: an emerging coronavirus that causes a global threat. Int J Biol Sci. 2020;16(10):1678‐1685. 32226285 10.7150/ijbs.45053 PMC7098030

[2] Singh DR , Sunuwar DR , Shah SK , et al. Impact of COVID‐19 on health services utilization in Province‐2 of Nepal: a qualitative study among community members and stakeholders. BMC Health Serv Res. 2021;21(1):174. 33627115 10.1186/s12913-021-06176-y PMC7903406

[3] Abhilash KP . Alpha, delta and now Omicron: when will the COVID‐19 pandemic end? Curr Med Issues. 2021;19(4):221.

[4] Rayamajhee B , Pokhrel A , Syangtan G , et al. How well the government of Nepal is responding to COVID‐19? An experience from a resource‐limited country to confront unprecedented pandemic. Front Public Health. 2021;9:597808. 33681124 10.3389/fpubh.2021.597808 PMC7925847

[5] Duch R , Roope LSJ , Violato M , et al. Citizens from 13 countries share similar preferences for COVID‐19 vaccine allocation priorities. Proc Natl Acad Sci USA. 2021;118(38):e2026382118. 34526400 10.1073/pnas.2026382118 PMC8463843

[6] Sharma K , Banstola A , Parajuli RR . Assessment of COVID‐19 pandemic in Nepal: a lockdown scenario analysis. Front Public Health. 2021;9:599280. 33898371 10.3389/fpubh.2021.599280 PMC8060478

[7] Basnet BB , Bishwakarma K , Pant RR , et al. Combating the COVID‐19 pandemic: experiences of the first wave from Nepal. Front Public Health. 2021;9:613402. 34322466 10.3389/fpubh.2021.613402 PMC8310916

[8] Talic S , Shah S , Wild H , et al. Effectiveness of public health measures in reducing the incidence of covid‐19, SARS‐CoV‐2 transmission, and covid‐19 mortality: systematic review and meta‐analysis. BMJ. 2021;375:e068302. 34789505 10.1136/bmj-2021-068302 PMC9423125

[9] Ministry of Health and Population, Nepal . Covid‐19 dashboard. 2022. Accessed March 3, 2022. https://covid19.mohp.gov.np/

[10] Fetzer TR , Witte M , Hensel L , et al. Global Behaviors and Perceptions at the Onset of the COVID‐19 Pandemic. National Bureau of Economic Research; 2020.

[11] Thapa M . Government's Response During COVID‐19 Pandemic in Nepal . Munich Personal RePEc Archive; 2021. Working paper 111666.

[12] Thunström L , Ashworth M , Shogren JF , Newbold S , Finnoff D . Testing for COVID‐19: willful ignorance or selfless behavior? Behavioural Public Policy. 2021;5(2):135‐152.

[13] Jimenez ME , Rivera‐Núñez Z , Crabtree BF , et al. Black and Latinx community perspectives on COVID‐19 mitigation behaviors, testing, and vaccines. JAMA Netw Open. 2021;4(7):e2117074. 34264327 10.1001/jamanetworkopen.2021.17074 PMC8283554

[14] Tao R , Downs J , Beckie TM , Chen Y , McNelley W . Examining spatial accessibility to COVID‐19 testing sites in Florida. Ann GIS. 2020;26(4):319‐327.

[15] Subedi D , Pantha S , Subedi S , et al. Perceptions towards COVID‐19 vaccines and willingness to vaccinate in Nepal. Vaccines. 2021;9(12):1448. 34960194 10.3390/vaccines9121448 PMC8703692

[16] Poudel A . Nepal begins first phase of COVID‐19 vaccination drive. *The Kathmandu Post*. 2021.

[17] WHO . 40% of Nepal's total population now fully vaccinated against COVID‐19. 2022. Accessed February 13, 2022. https://www.who.int/nepal/news/detail/16-01-2022-40-of-nepal-s-total-population-now-fully-vaccinated-against-covid-19

[18] Panigrahi SK , Majumdar S , Galhotra A , Kadle SC , John AS . Community based management of COVID‐19 as a way forward for pandemic response. Front Public Health. 2021;8:1010. 10.3389/fpubh.2020.589772 PMC7838456 33520913

[19] Narayana G , Pradeepkumar B , Ramaiah JD , Jayasree T , Yadav DL , Kumar BK . Knowledge, perception, and practices towards COVID‐19 pandemic among general public of India: a cross‐sectional online survey. Curr Med Res Pract. 2020;10(4):153‐159. 32839725 10.1016/j.cmrp.2020.07.013 PMC7372279

[20] Mannan DKA , Mannan KA . Knowledge and perception towards novel coronavirus (COVID 19) in Bangladesh. Int Res J Bus Social Sci. 2020;6(2).

[21] Hennink M , Hutter I , Bailey A . Qualitative Research Methods. Sage; 2020.

[22] Booth A , Hannes K , Harden A , Noyes J , Harris J , Tong A . COREQ (consolidated criteria for reporting qualitative studies). In: Moher D , Altman DG , Schulz KF , Simera I , Wager E , eds. Guidelines for Reporting Health Research: A User's Manual. Wiley; 2014:214‐226.

[23] Legard R , Keegan J , Ward K . In‐depth interviews. In: Richie J , Lewis J , eds. Qualitative Research Practice: A Guide for Social Science Students and Researchers. Sage; 2003:138‐169.

[24] Scanlan CL . Preparing for the Unanticipated: Challenges in Conducting Semi‐Structured, In‐Depth Interviews. SAGE Publications Ltd; 2020.

[25] Clarke V , Braun V , Hayfield N . Thematic analysis. In: Smith JA , ed. Qualitative Psychology: a Practical Guide to Research Methods. SAGE Publications; 2015:222‐248.

[26] Leech NL , Onwuegbuzie AJ . Beyond constant comparison qualitative data analysis: using NVivo. Sch Psychol Q. 2011;26(1):70‐84.

[27] Parajuli SB , Kuikel J , Lama S , Heera K . Efforts and challenges in the COVID‐19 mitigation in Nepal. Europasian J Med Sci. 2020;2(2):132‐134.

[28] Khankeh H , Farrokhi M , Roudini J , et al. Challenges to manage pandemic of coronavirus disease (COVID‐19) in Iran with a special situation: a qualitative multi‐method study. BMC Public Health. 2021;21(1):1919. 10.1186/s12889-021-11973-5 34686165 PMC8532398

[29] BC UB , Pokharel S , Munikar S , et al. Anxiety and depression among people living in quarantine centers during COVID‐19 pandemic: a mixed method study from western Nepal. PLoS One. 2021;16(7):e0254126. 34242319 10.1371/journal.pone.0254126 PMC8270129

[30] Chew C‐C , Lim X‐J , Chang C‐T , Rajan P , Nasir N , Low W‐Y . Experiences of social stigma among patients tested positive for COVID‐19 and their family members: a qualitative study. BMC Public Health. 2021;21(1):1623. 10.1186/s12889-021-11679-8 34488693 PMC8419662

[31] Ayenew B , Pandey D . Challenges and opportunities to tackle COVID‐19 spread in Ethiopia. J PeerScientist. 2020;2(2):e1000014.

[32] Tamang P , Mahato P , Shahi P , Simkhada P , Van Teijlingen E , Amgain K . COVID‐19 quarantine: a key part of prevention in Nepal. J Karnali Acad Health Sci. 2020;3(1):1‐14

[33] Nafees M , Khan F . Pakistan's response to COVID‐19 pandemic and efficacy of quarantine and partial lockdown: a review. Electron J Gen Med. 2020;17(2):em240.

[34] Kansakar S , Dumre SP , Raut A , Huy NT . From lockdown to vaccines: challenges and response in Nepal during the COVID‐19 pandemic. Lancet Respir Med. 2021;9(7):694‐695. 33932347 10.1016/S2213-2600(21)00208-3 PMC8081397

[35] Jewkes SV , Zhang Y , Nicholl DJ . Nosocomial spread of COVID‐19: lessons learned from an audit on a stroke/neurology ward in a UK district general hospital. Clin Med. 2020;20(5):e173‐e177. 10.7861/clinmed.2020-0422 PMC7539735 32719035

[36] Sah R , Khatiwada AP , Shrestha S , et al. COVID‐19 vaccination campaign in Nepal, emerging UK variant and futuristic vaccination strategies to combat the ongoing pandemic. Travel Med Infect Dis. 2021;41:102037. 33781945 10.1016/j.tmaid.2021.102037 PMC7997904

[37] Su Z , Wen J , McDonnell D , et al. Vaccines are not yet a silver bullet: the imperative of continued communication about the importance of COVID‐19 safety measures. Brain Behav Immun Health. 2021;12:100204. 33495754 10.1016/j.bbih.2021.100204 PMC7817456

[38] Abbas J , Wang D , Su Z , Ziapour A . The role of social media in the advent of COVID‐19 pandemic: crisis management, mental health challenges and implications. Risk Manag Healthc Policy. 2021;14:1917‐1932. 10.2147/RMHP.S284313 34012304 PMC8126999

